# Regulation of Cholesterol and Triglyceride Metabolism by Fatty acid Ethanolamides

**DOI:** 10.1007/s11883-026-01417-z

**Published:** 2026-05-02

**Authors:** Sean S. Davies, Reza Fadaei

**Affiliations:** 1 Department of Pharmacology, School of Medicine Basic Sciences, Vanderbilt University, 556B Robinson Research Building, Nashville, TN 37232-6602 USA; 2 Vanderbilt Institute of Chemical Biology, Vanderbilt University, Nashville, TN 37232 USA; 3 Nashville, USA

**Keywords:** *N*-acyl-ethanolamine, *N*-acyl-phosphatidylethanolamine, metabolic regulation, NAPE-PLD, PPARα, cardiometabolic diseases

## Abstract

**Purpose of Review:**

This paper reviews the existing literature supporting a role for fatty acid ethanolamides including oleoylethanolamide and palmitoylethanolamide in regulating cholesterol and triglyceride metabolism and the mechanisms underlying these effects.

**Recent Findings:**

Deletion of various fatty acid ethanolamide biosynthesis genes in cellular models and small animal models have provided additional evidence that endogenous biosynthesis of fatty acid ethanolamides regulates cholesterol and triglyceride metabolism, while new clinical trials using oleoylethanolamide and palmitoylethanolamide support their therapeutic potential to normalize lipid levels in individuals with cardiometabolic disease.

**Summary:**

Fatty acid ethanolamides are biosynthesized in the intestine, liver, and adipose tissue in response to metabolic stimuli like fasting and feeding. These fatty acid ethanolamides then act via receptors including PPARα, GPR119, and GPR55 to regulate cholesterol and triglyceride levels and promote the resolution of inflammation, thereby protecting against cardiometabolic diseases including metabolic-dysfunction associated steatotic liver disease and atherosclerotic cardiovascular disease.

**Graphical Abstract:**

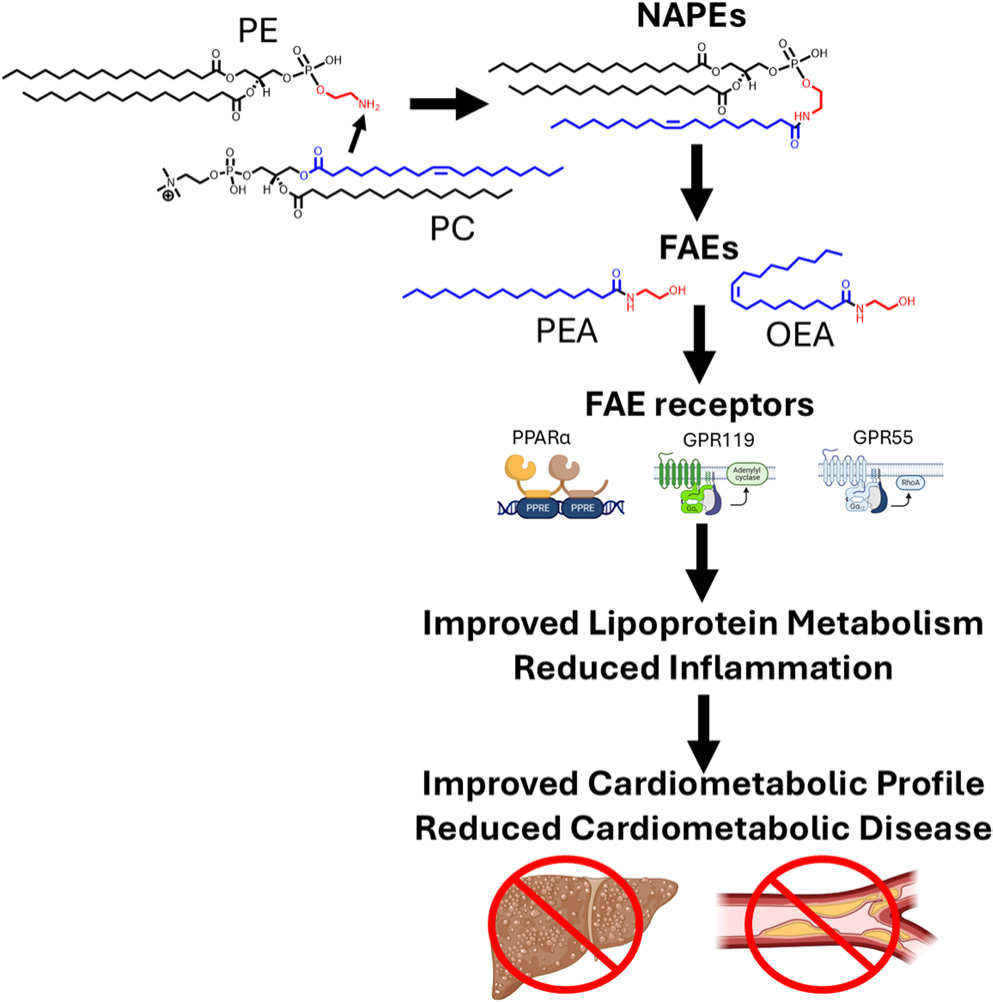

## Introduction

**Fig. 1:**
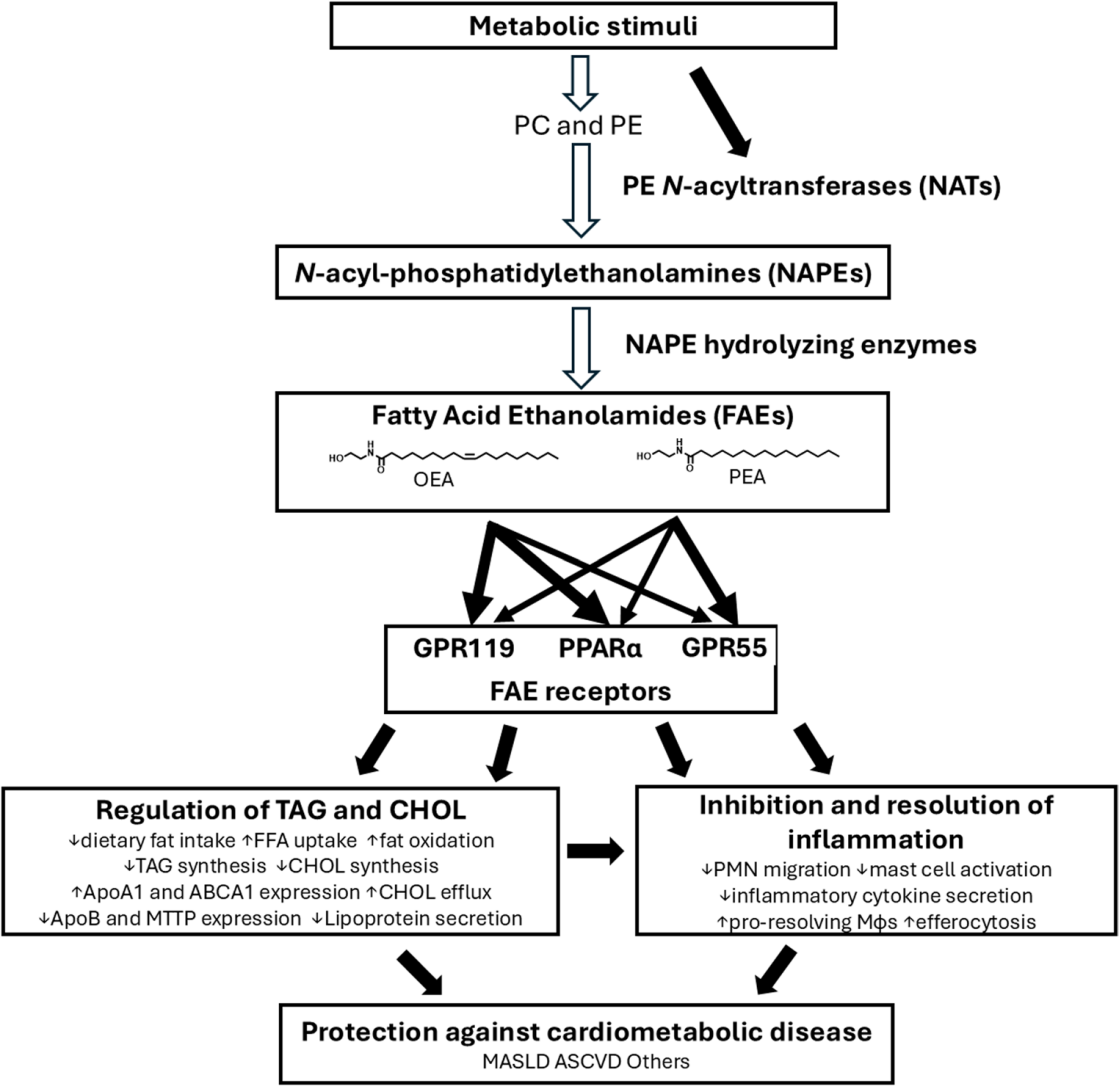
Biosynthesis of fatty acid ethanolamides protects against cardiometabolic diseases. Metabolic stimuli that cause an increase in free fatty acids (FFA) and subsequent increases in phosphatidylcholine (PC) and phosphatidylethanolamine (PE) trigger increased formation of fatty acid ethanolamides (FAEs) including oleoylethanolamide (OEA) and palmitoylethanolamide (PEA). These metabolic stimuli may increase the activity of the N-acyltransferases that form NAPEs. OEA and PEA then act on receptors including PPARα, GPR119, and GPR55 to regulate triglyceride (TAG) and cholesterol (CHOL) metabolism and inflammation. The integrated effects of FAE receptor activation protect against cardiometabolic diseases including metabolic-dysfunction associated steatotic liver disease (MASLD) and atherosclerotic cardiovascular disease (ASCVD). Hollow arrows indicate biochemical synthesis pathways and filled arrows represent signaling pathways,

In 2001, Rodríguez de Fonseca et al. reported that ingestion of dietary fat triggered oleoylethanolamide (OEA) production in the small intestine and that OEA inhibited food intake [1]. In the 25 years since this discovery, fatty acid ethanolamides (FAEs) including OEA and palmitoylethanolamide (PEA) have emerged as key bioactive lipids produced in the intestine, liver, and adipose tissues that signal through PPARα, GPR119, and GPR55 to regulate the metabolism of triglycerides and cholesterol and promote the resolution of inflammation that may therefore protect against the development and progression of cardiometabolic diseases including atherosclerosis (Fig. 1). This review will summarize the key studies elucidating the mechanisms by which FAEs act and emerging pre-clinical and clinical evidence supporting their therapeutic potential as lipid lowering and anti-atherosclerotic agents. It will also highlight key gaps in our current knowledge including how the biosynthesis of these bioactive lipids is regulated and why FAE biosynthesis becomes dysregulated during development of cardiometabolic diseases.

### Fatty acid Ethanolamide Biosynthesis

**Fig. 2:**
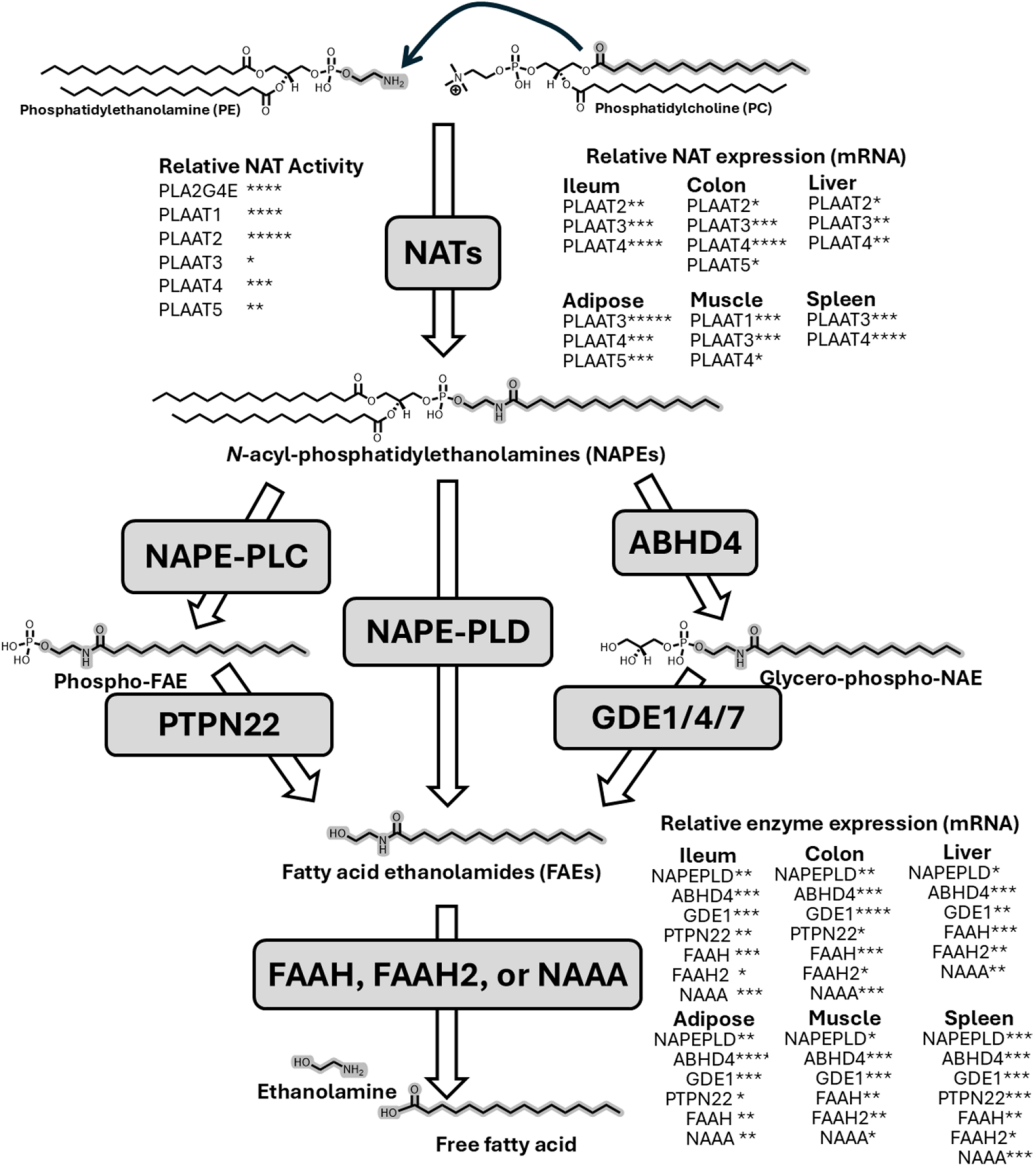
Distribution and relative expression of the enzymes of fatty acid ethanolamide biosynthesis and catabolism in metabolically active tissue. In humans, six known PE N-acyltransferases (NATs) biosynthesize NAPEs by transferring an acyl chain from phosphatidylcholine (PC) to the nitrogen of phosphatidylethanolamine (PE). The atoms of each molecule which eventually form the final FAE are highlighted in grey. The relative intrinsic NAT activity of each enzyme is illustrated by number of + symbols based on Uyuma et al. [2] and Ogura et al. [3]. Three independent pathways convert NAPEs to FAEs. FAEs are inactivated by catabolism by Fatty Acid Amide Hydrolase (FAAH), FAAH2, or N-acylethanolamine hydrolyzing acid amidase (NAAA). The relative mRNA expression (median transcripts per million, TPM) of each gene encoding a biosynthetic or catabolizing enzyme is indicated by number of * symbols as follows: not shown TPM ≤ 1; *1 < TPM ≤ 5; **5 < TPM ≤ 15; ***15 < TPM ≤ 45; ****45 < TPM ≤ 145; *****TPM > 145, based on data exported from the Genotype-Tissue Expression (GTEx) Portal on 11/02/2025. The GTEx project is supported by the Common Fund of the Office of the Director of the National Institutes of Health, and by NCI, NHGRI, NHLBI, NIDA, NIMH, and NINDS

Besides OEA and PEA, other major species of FAE (also known as *N*-acyl-ethanolamines) include stearoylethanolamide (SEA), linoleoylethanolamide (LEA), and arachidonoylethanolamide (AEA, also known as anandamide). The overall framework for FAE biosynthesis has been elucidated, but important details related to the regulation of their biosynthesis in response to stimuli remain uncharacterized. In vertebrates, biosynthesis of FAEs requires at least two steps: the formation of *N*-acyl-phosphatidylethanolamines (NAPEs) and then enzymatic hydrolysis of these NAPEs to generate FAEs. Phosphatidylethanolamine *N*-acyltransferases (NATs) transfer an *O*-acyl chain from phosphatidylcholine (PC) or phosphatidylethanolamine (PE) to the headgroup nitrogen of an acceptor PE to form NAPEs (Fig. 2). Humans express at least six different NATs: a family of five calcium-independent NATs, the phospholipase A/acyl transferases (PLAAT-1 to -5) that differ significantly in terms of their NAT activity [2] and a calcium-dependent NAT, phospholipase A_2_ group 4 epsilon (PLA24ε) [3]. Three known pathways hydrolyze NAPEs to FAEs. NAPE-hydrolyzing phospholipase D (NAPE-PLD) hydrolyzes the distal phosphodiester bond of NAPE to directly generate FAE and phosphatidic acid. In mice, genetic deletion of *Napepld* globally or in specific tissues reduced levels of individual FAEs by 20–70% [4–8], revealing that NAPE-PLD-independent pathways also contributed to FAE biosynthesis. Subsequently, ABDH4 was shown to hydrolyze NAPEs to form glycerophospho-*N*-acyl-ethanolamines [9] which can then be hydrolyzed by GDE1 to form FAE [10]. NAPE were also shown to be hydrolyzed to phospho-FAEs by an as yet unidentified NAPE-hydrolyzing phospholipase C, with the phospho-FAE then hydrolyzed by PTPN22 to form FAE [11]. The intestine, liver, adipose tissue, and skeletal muscle of humans each express multiple NATs and NAPE-hydrolyzing enzymes (Fig. 2).

Individual FAEs vary significantly from one another in their levels in each tissue and these levels differentially change in response to metabolic stimuli. For instance, Petersen et al. reported that intestinal NAEs in fasted rats were as follows: OEA 1.4 ± 0.4, PEA 3.9 ± 0.22, SEA 4.2 ± 0.15, LEA 2.8 ± 0.4, and AEA 0.46 ± 0.15 pmol / µmol phospholipid [12]. Refeeding increased levels of intestinal OEA 5.6-fold, PEA 2.0-fold, SEA 1.1-fold, LEA 11.7-fold, and reduced levels of AEA 2.6-fold [12]. The relatively low levels of AEA compared to other FAEs in peripheral tissue likely results from all NATs preferentially utilizing *O*-acyl chains from the *sn-1* position of PC where the acyl group is typically palmitate, stearate, or oleate, rather than arachidonate. Differences between individual FAE levels is also the result of differential expression of the two major FAE catabolizing enzymes, Fatty Acid Amide Hydrolyase (FAAH) and *N*-acyl-ethanolamine hydrolyzing acid amidase (NAAA) in various tissues (Fig. 2). Unlike rodents, humans also express a second FAAH enzyme, FAAH-2. FAAH preferentially catabolizes AEA over OEA and PEA [13], while NAAA preferentially catabolizes saturated FAEs like PEA [14]. However, the extent to which individuals NATs, NAPE-hydrolyzing enzymes, and FAE catabolizing enzymes contribute to individual FAE levels in each tissue is poorly characterized. In macrophages, LPS appears to stimulate AEA biosynthesis by upregulating the NAPE-PLC/PTPN22 pathway [11] while inhibiting PEA biosynthesis by reducing NAPE-PLD expression [15]. Because individual FAE species differ in their physiological effects, a better understanding of how levels of each FAE are differentially regulated may be critical to a complete understanding of how FAEs regulate triglyceride and cholesterol metabolism.

### Changes in Intracellular Levels of free Fatty Acids Triggers FAE Biosynthesis

Feeding-induced increases in intestinal FAEs have been observed in all vertebrate species where it has been examined [1, 12, 16–19]. Fat ingestion drives the feeding-induced increase in intestinal FAEs [20, 21] with free fatty acids (FFAs) released from ingested triglycerides (TAGs) being transferred into enterocytes by the fatty acid translocase CD36 and the newly acquired FFAs incorporated into NAPEs [20], presumably after the FFA is first incorporated into PCs. In liver and adipose tissue, FAE levels are at their apex during fasting and at their nadir after feeding [16, 22], consistent with fasting increasing intracellular FFA in adipocytes and hepatocytes. Thus, rising intracellular FFA levels trigger FAE production, positioning them to initiate functional responses that help restore lipid homeostasis.

Increased intestinal biosynthesis of *N-*oleoyl-PE, *N*-linoleoyl-PE, and *N*-palmitoyl-PE appear to be required for the increases in their respective FAEs during fasting and refeeding [12, 16]. Unfortunately, the specific NATs in the mammalian intestine, liver, and adipose tissue that generate NAPEs in response to rising FFAs levels are unknown. We have shown that genetic deletion of *plaatl1* in zebrafish blocks the feeding-induced rise in intestinal NAPEs and FAEs and leads to weight gain and fat accumulation [19]; however, *plaat1l1* is not a direct ortholog of human *PLAAT1* or mouse *Plaat1*, so another PLAAT/Plaat may be responsible in mammals. Furthermore, precisely how elevated FFA levels trigger increased NAPE biosynthesis remains poorly understood. The substrates for NATs are PC and PE, not FFA or FFA-CoA [2, 23]. Even though FFA influxes increase PC and PE synthesis, both phospholipids would already seem to be available to NATs at saturating concentrations as they are the primary components of membranes and are present at concentration far above their critical micellar concentration. Thus, the mechanism(s) whereby intracellular FFA levels increase NAPE/FAE biosynthesis still requires elucidation.

### Deletion of Endogenous FAE Biosynthetic Enzymes Induces Dyslipidemia.

Support for the notion that FAE biosynthesis is required for appropriate regulation of triglyceride (TAG) and cholesterol (CHOL) levels comes from studies where a key FAE biosynthetic enzyme, NAPE-PLD, has been genetically deleted in three key sites of lipid homeostasis—the liver, intestine, and adipose tissue. Liver-specific deletion of *Napepld* (*Napepld*^*ΔHep*^) only reduced liver levels of OEA and LEA ~ 20% and did not change levels of AEA or PEA compared to wild-type (WT) mice [6]. Even so, *Napepld*^*ΔHep*^ mice had increased total body fat mass compared to WT mice [6]. Average lipid droplet size was increased in the liver of *Napepld*^*ΔHep*^ mice, although total liver fat mass did not. *Napepld*^*ΔHep*^ mice had significant reductions in both liver bile acid and oxysterols, but not CHOL [6]. Plasma total or lipoprotein CHOL were not reported, but *Napepld*^*ΔHep*^ showed greater insulin resistance [6], which is often correlated with an increase in plasma TAG to HDL-C ratio.

More compelling evidence for FAE biosynthesis regulating lipid homeostasis comes from mice where *Napepld* is specifically deleted in intestinal epithelial cells (*Napepld*^*ΔIEC*^ mice). Intestinal OEA, PEA, SEA, and AEA levels are reduced by ~ 50% in *Napepld*^*ΔIEC*^ mice compared to WT mice [4]. On high-fat diet (HFD), *Napepld*^*ΔIEC*^ mice show increased liver TAGs and lipid droplet size, but no significant changes in liver CHOL levels [4]. Immediately following an oral lipid challenge, *Napepld*^*ΔIEC*^ mice show modest increases in plasma TAGs, but not FFAs, compared to WT mice [4]. On first exposure to HFD, *Napepld*^*ΔIEC*^ mice also eat more than WT mice and after 8 weeks of HFD *Napepld*^*ΔIEC*^ mice show significantly increased adipose tissue weight and total body weight compared to WT mice [4]. *Napepld*^*ΔIEC*^ mice showed no significant changes in insulin resistance and glucose tolerance [4].

The most compelling evidence for FAE biosynthesis regulating lipid homeostasis comes from studies of mice with adipose tissue-specific deletion of *Napepld* (*Napepld*^*ΔAdipo*^ mice). Adipose OEA, PEA, and SEA levels in *Napepld*^*ΔAdipo*^ mice are ~ 50% of those found in WT mice, with no change in AEA levels [5]. Even when fed low-fat diet, *Napepld*^*ΔAdipo*^ mice had 20–25% higher plasma TAG and CHOL than WT mice [5]. Food intake in *Napepld*^*ΔAdipo*^ mice was not increased compared to WT mice on either a low-fat or high-fat diet; nevertheless, total body weight increased ~ 25%, total body fat increased ~ 50%, and glucose tolerance was impaired [5]. Of note, adipose-specific deletion of *Abhd4* in mice did not alter FAE levels in adipose tissue or increase adiposity or total body weight compared to WT mice [24].

Determining the full extent to which endogenous FAE biosynthesis regulates lipid homeostasis will require a more complete ablation of FAE biosynthesis, such as by concomitantly deleting NAT genes (i.e. Plaat1-5 and Pla2g4e) and NAPE-PLD.

### Administering FAEs Normalizes Triglyceride and Cholesterol Homeostasis and Inhibits Atherosclerosis Progression

Further evidence for FAEs playing an important role in the regulation of TAGs and CHOL and in the prevention of cardiometabolic diseases including metabolic-dysfunction associated steatotic liver disease (MASLD) and atherosclerotic cardiovardiovascular disease (ASCVD) comes from studies where various FAEs have been administered to rodents or humans. In mice, intraperitoneal (i.p.) administration of OEA administration (5 mg/kg/day) reduced tissue levels of TAGs in liver and adipose tissues [25], while oral OEA administration (100 mg/kg/day) reduced plasma TAG by 59% and plasma CHOL by 17% [26]. In Zucker obese rats, i.p. OEA administration (5 mg/kg/day) reduced both TAG and CHOL levels in serum and hepatocytes [27], while in Sprague-Dawley rats fed a high-fat diet (HFD), it reduced both their plasma TAG and their plasma CHOL by > 50%, lowered circulating levels of liver enzymes and inflammatory cytokines, and reduced hepatosteatosis [28]. In rats given valproic acid to induce steatohepatitis, i.p. OEA administration (10 mg/kg/day) reduced serum TAG, low density lipoprotein-cholesterol (LDL-C), high density lipoprotein-cholesterol (HDL-C), and total CHOL, and reversed their steatohepatitis as well as reduced ALT/AST levels [29]. Other FAEs besides OEA also exert clear lipid-lowering effects. Administering PEA (30 mg/kg s.c. daily) to ovariectomized rats reduced their serum CHOL levels, but not serum TAG levels [30]. This treatment also reduced food intake, body weight and fat mass, and improved glucose tolerance [30]. In mice fed HFD, oral PEA administration (30 mg/kg/day) reduced serum levels of TAG, total CHOL, liver enzymes, insulin and glucose, and resulted in improved glucose tolerance and insulin sensitivity [31]. In Spraque-Dawley rats previously made obese by feeding a cafeteria diet for 12 weeks, administering LEA (10 mg/kg i.p.) for 14 days resulted in large reductions in plasma TAGs and CHOL, as well as uric acid, AST, IL-6, and TNFα [32].

Pilot clinical trials show similar lipid-lowering effects. Giving oral OEA (300 mg/day) to twenty stroke patients reduced their plasma TAG, HDL-C, total CHOL, urea, IL-6, and hsCRP compared to patients receiving placebo [33]. Giving oral OEA (250 mg/day) to thirty obese MASLD patients reduced their plasma TAG, BMI and fat mass compared to similar patients receiving placebo [34]. Oral OEA (250 mg/day) significantly reduced serum TAG levels in thirty obese individuals compared to placebo-treated individuals, but did not significantly alter their LDL-C, HDL-C or total CHOL [35]. Giving PEA (700 mg daily, orally) to 29 obese adults reduced plasma TAG and IL-2 levels compared to placebo treatment (15 adults), without changing HDL-C, LDL-C, fasting glucose, or various other cytokines and markers of inflammation [36]. While these small-scale studies support FAEs playing a key role in the regulation of lipid homeostasis in humans, whether pharmacologic administration of FAEs is an effective clinical treatment for humans with significant dyslipidemia awaits large scale randomized control trials.

A major goal of cholesterol-lowering therapies is to inhibit the development of ASCVD, and several studies in mice support the potential efficacy of administering FAEs in this regard. For instance, administration of OEA (5 mg/kg daily i.p.) visibly reduced the amount of atherosclerotic plaque in balloon-aorta denudation mice or in *Apoe*^*−/−*^ mice fed a Western diet, although this was not quantified [37]. Administration of PEA (3 mg/kg daily i.p.) reduced atherosclerotic lesion area in *Apoe*^*−/−*^ mice when PEA administration began with the start of Western diet [38]. If mice were fed Western diet for 12 weeks prior to beginning PEA administration, PEA did not reduce atherosclerotic lesion area but did reduce necrotic core area and increased fibrous cap thickness [38], consistent with plaque stabilization. We showed that raising tissue FAE level by orally administering bacteria genetically engineered to secrete NAPEs significantly reduced serum CHOL levels and atherosclerotic lesion necrotic core area in *Ldlr*^*−/−*^ mice fed a Western diet [39]. Despite these promising results in mouse models, no randomized clinical trials examining the efficacy of PEA or OEA in the treatment of ASCVD have been undertaken.

### Potential Mechanisms for the Anti-Atherosclerotic Effects of FAEs

Many of the protective effects of FAEs against cardiometabolic diseases can be rationalized by their activation of three key receptors, PPARα, GPR119, and GPR55 (Fig. 1) as synthetic agonists of these receptors reduce fat intake, increase fat oxidation, reduce fat synthesis, decrease secretion of ApoB-containing lipoproteins, reduce inflammation, and enhance the resolution of inflammation.

Peroxisome Proliferator-Activated Receptor alpha (PPARα) is a nuclear hormone receptor that heterodimerizes with the retinoid X receptor to drive expression of a large number of genes related to lipid metabolism. OEA is one of the most potent endogenous agonists of PPARα (EC_50_ 120 nM) [40], with PEA also being a PPARα agonist (EC₅₀ 3.1 µM) [41] (Fig. 1). AEA has been reported to either not activate PPARα [41] or to do so only at high concentration [42]. While several actions of SEA and LEA require PPARα, to best of our knowledge their binding affinity for PPARα has not been reported. FAEs also activate G-protein coupled receptors including GPR119 which primarily couples to Gα_s_, GPR55 that primarily couples to Gα_12/13_, and endocannabinoid receptor-1 and − 2 (CNR1 and CNR2, respectively) that primarily couple to Gα_i/o_. OEA was the first endogenous GPR119 agonist identified (EC_50_ 0.2–3.2 µM, depending on downstream readout) [43–45]. To the best of our knowledge, concentration response curves for GPR119 activation by other FAEs have not been reported, but in cAMP-reporter based assay, 30 µM of OEA, PEA, SEA, and AEA were shown to induce 37-fold, 17-fold, 5-fold, and 2-fold increases in reporter activity, respectively [43]. PEA is a highly potent GPR55 agonist (EC_50_ 4 nM), with AEA and OEA being somewhat less potent (EC_50_ 18 nM and 440 nM, respectively) [14]. However, not all studies have found PEA or other FAEs to activate GPR55 signaling [46, 47] and GPR55 activation by FAE may require concomitant integrin clustering [48]. Activation of CNR1 has many well-established neurological effects including increasing appetite [49], while activation of CNR2 modulates immune cell function. AEA is a robust partial agonist of CNR1 (EC_50_ 31 nM) and CNR2 (EC_50_ 27 nM) [50, 51]. Importantly, saturated and monounsaturated FAEs such as PEA, SEA, and OEA do not have any meaningful efficacy for CNR1 or CNR2 [50, 52].

Administering OEA significantly reduces food intake [1]. Since intestinal OEA biosynthesis is triggered by fat ingestion, OEA’s satiation effect presumably helps prevent an overload of dietary fats. The satiating effects of OEA require *Ppara* [40]. PEA, SEA, and LEA reduce food intake by rodents less potently than OEA [1, 53–55], consistent with their lower potency as PPARα agonists. AEA promotes food intake [1], consistent with agonism of CNR1 being the primary action of AEA [49] and highlights to importance of the finding that intestinal AEA levels drop in response to fat ingestion in contrast to other FAEs. Activation of PPARα by OEA induces satiation and satiety by multiple mechanisms that require oxytocin or histamine [56–60]. In addition to its effects on PPARα, OEA induces L-cell secretion of GLP-1 via activation of GPR119 [61] and OEA associates with GLP-1 to increase its potency at GLP-1R [62]. However, deletion of *Gpr119* does not block inhibition of food intake by OEA [63], and OEA-induced satiation does not require secretion of GLP-1 or other peptide hormones such as CCK, PYY, or ghrelin [64]. Thus, activation of PPARα appears to be the primary mechanism for FAEs to inhibit fat ingestion.

As noted in prior sections, administration of OEA markedly reduces tissue TAG levels. This effect is lost in *PPARα*^*−/−*^ mice [25], suggesting that activation of PPARα is required for the lipid lowering effect of OEA and likely other FAEs. PPARα agonists like fenofibrates are well-characterized to induce genes that enhance fatty acid oxidation and promote lipoprotein catabolism including CD36, acyl-CoA synthetase, FABP1, CPT1A and CPT2, ACAD-M, -L, and -VL, ACOX, LPL, FGF21, and LPIN2 [65]. In humans, PPARα agonists increase expression of *APOA1* (the major apolipoprotein of HDL), but in mice they inhibit *Apoa1* expression, due to an altered PPAR responsive element in the *Apoa1* gene [66]. Also in humans but not mice, PPARα agonists increase cholesterol efflux from macrophages (the key step in reverse cholesterol transport) by inducing macrophage expression of ABCA1 [66, 67], which transfers CHOL to lipid-poor HDL particles.

Activation of GPR119 also exerts lipid lowering effects. For instance, overexpression of GPR119 lowered the total and free CHOL levels in THP-1 macrophages both before and after cholesterol loading [68]. GPR119 overexpression increased expression of ABCA1 and this effect required GLP1R [68], consistent with GPR119 activation inducing GLP-1 secretion to exert its downstream effects. Lentivirus-mediated overexpression of GPR119 in *Apoe*^*−/−*^ mice reduced serum TAG 28% and increased serum ApoA1 levels 40%, but did not alter serum CHOL levels or ApoB levels; nevertheless, *Gpr119* overexpression did reduce liver CHOL levels by 27% and TAG levels by 28%, and increased liver ABCA1 and SRB1 expression without altering HMGS, HMGCR, LDLR, or ABCG1 expression and reduced atherosclerotic lesion area [68]. Similarly, treating spontaneously hyperlipidemic mice with the GPR119 agonist JTP-109,192 significantly reduced their total plasma CHOL and their atherosclerotic lesion area [69].

Many of the cellular effects of FAEs mirror those of synthetic PPARα and GPR119 agonists. OEA stimulates lipolysis and induces increased fat oxidation in skeletal muscle, adipocytes, and hepatocytes [25]. OEA also increases CD36 expression and FFA uptake in intestinal cells and adipocytes [70]. Treating HepG2 hepatocyte cells with very high concentrations of OEA (50 µM) increased expression of CPT1A and EHCS1 in PPARα-dependent manner and decreased expression of SCD1 in a PPARα-independent manner [28]. OEA decreased ApoB secretion by human hepatocyte cell lines (HepG2 and Huh-7) in a concentration dependent manner, with inhibition observed even at 20 µM OEA [71]. OEA also inhibited MTP activity and reduced total VLDL secreted from the Huh-7 cells. Similar inhibition by OEA of TAG and phospholipid secretion and MTP activity was seen in cultured primary hepatocytes from wild-type mice. OEA did not reduce TAG and phospholipid secretion by primary hepatocytes derived from *Ppara*^*−/−*^ mice, although it is worth noting that *Ppara*^*−/−*^ hepatocytes already have significantly lower lipid secretion compared to wild-type hepatocytes [71].

Differentiation of human enterocyte Caco2 cell line increases their expression of *NAPEPLD* while modestly decreasing expression of *GDE1* and *ABHD4* [72]. NAPE-PLD is the major NAPE-hydrolyzing enzyme producing FAEs in differentiated Caco2, as CRISPR/cas9 mediated knockdown of *NAPEPLD* in these cells reduced intracellular FAE levels > 70% [72]. *NAPEPLD* knockdown also markedly reduced expression of *APOA4*, *APOC3*, *ABHD4*, and *FAAH* and increased expression of *APOB*, *MTTP*, *FABP1*, *FABP2*, *CPT1A*, and especially *CD36*. *NAPEPLD* knockdown increased secretion of TAG and CHOL containing lipoproteins, especially in lipid-rich culture conditions, and thereby lowered intracellular levels of TAG, CHOL, and FFA [72]. Of note, vertical sleeve gastrectomy (VSG) increases duodenal levels of OEA and normalizes circulating TAG and CHOL levels in mice fed HFD; however, VSG still normalizes lipid levels in *Ppara*^*−/−*^ and *Gpr119*^*−/−*^ mice, suggesting that OEA signaling through these receptors is not required for the rescue of lipid homeostasis induced by VSG [73].

*Abhd4* knockdown (KD) in the mouse preadipocyte 3T3L1 cell line suggest that FAE biosynthesis downregulates FFA and CHOL synthesis by these cells as KD reduced FAE levels and markedly increased rates of de novo fatty acid, cholesterol, and phospholipid synthesis from [^3^H]Acetate [24]. Furthermore, primary adipocytes from *Abhd4*^*−/−*^ mice showed greater TAG accumulation than those from WT mice [24]. However, *Abhd4*^*−/*−^ mice showed no increases in adiposity or total body weight compared to WT mice, either when fed a low-fat or high-fat diet [24] and did not show any difference in the levels of OEA, PEA, and AEA in their adipose tissue, suggesting that in whole animals, the Abhd4 pathway is not a significant of FAEs in adipose tissue.

Although this review focuses on the regulation of lipid homeostasis, FAEs do exert anti-atherosclerotic effects that may be independent of their lipid-lowering effects. Synthetic agonists of PPARα [74, 75], GPR55 [76–78], and GPR119 [79–81] promote anti-inflammatory and inflammation resolving effects, providing a theoretical framework to understand how FAEs could promote similar effects. PEA has well established mast cell stabilizing effects [82, 83] and inhibits PMN migration and edema induced by inflammatory agents like carrageenan and substance P, with these protective effects being lost in PPARα^−/−^ mice [41, 82, 84, 85]. PEA also inhibits neuroinflammation and nociception (reviewed by Petrosino and Di Marzo) [86]. OEA inhibits inflammation in the collagen-induced arthritis mouse model by suppressing inflammatory cytokine secretion and Th_1_ and Th_17_ differentiation, while promoting T_reg_ differentiation and these effects are ablated in *Gpr119*^*−/−*^ mice [87]. Similar Gpr119-dependent anti-inflammatory effects of OEA were observed in mouse models of allergic asthma and atopic dermatitis [88]. OEA also polarizes macrophages away from an pro-inflammatory (M1-like) phenotype and towards a pro-resolving (M2-like) phenotype including by downregulating TNFα, IL1β, and IL6 and upregulating Arg1, YM1, and CD206 [89, 90]. These anti-inflammatory effects require PPARα. Another critical step in the resolution of inflammation is the clearance of apoptotic cells from the sites of inflammation by macrophages (i.e. efferocytosis). PEA enhances the ability of bone-marrow derived macrophages to carry out efferocytosis, regardless of whether these macrophages are polarized to M1-like or M2-like phenotype [38]. Interestingly, this effect is mediated in part by GPR55-dependent increases in the expression of the key efferocytosis receptor MerTK [38]. Small molecule activators of NAPE-PLD increase efferocytosis by cultured mouse primary macrophages and genetic deletion of *Napepld* or inhibition of NAPE-PLD reduce efferocytosis [91].

Despite these recent discoveries, significant unanswered questions remain about how FAEs exert their powerful lipid lowering and anti-atherosclerotic effects including to what extent loss of endogenous FAE biosynthetic enzymes such as NATs and NAPE-PLD exacerbate atherosclerosis in mouse models.

### FAE Biosynthesis Becomes Dysfunctional During Cardiometabolic Diseases

If FAE biosynthesis and signaling play a critical role in regulating lipid homeostasis, then presumably FAE biosynthesis initially increases to compensate for dietary lipid overload and other risk factors that drive development of cardiometabolic diseases but then this biosynthetic pathway becomes exhausted and dysfunctional. Studies to date support this notion, although the precise mechanisms for such changes remain unknown. Compared to mice fed standard diet, mice fed HFD show reduced levels of OEA and PEA (3-fold and 10-fold, respectively) in subcutaneous adipose tissue (SAT), but not visceral adipose tissue (VAT) [42]. Compared to *Apoe*^*−/−*^ mice fed standard diet, those fed a Western diet show reduced PEA levels in VAT, with no changes in aorta. In contrast, Western diet increases OEA levels in both VAT and aorta [92]. Fasted obese Zucker rats have markedly elevated levels of OEA and PEA in their duodenum compared to lean control rats, but reduced levels of OEA and PEA in their liver, SAT, VAT, pancreas, and brain [22]. Refeeding increases duodenal OEA levels in both lean and Zucker rats but decreases duodenal PEA levels in the Zucker rats [22]. Refeeding decreases both PEA and OEA levels compared to fasting in most other tissues of both lean and Zucker rats [22]. Feeding rats HFD leads to time-dependent decreases in intestinal levels of PEA, LEA, and OEA [53]. HFD also decreases intestinal *Napepld* expression and GDE1 activity without altering FAAH expression [53].

Studies in humans consistently find that cardiometabolic diseases are associated with altered FAE levels, although most of these studies have small sample sizes. For instance, Matias *et a*l found that levels of PEA in SAT, but not VAT, were reduced in obese human subjects (9 M/11F) compared to normal weight subjects (4 M/6F), while OEA levels were unchanged [42]. They also found that plasma PEA and OEA levels were ~ 50% higher in individuals with type 2 diabetes compared to control [42]. Quercioli et al. found that average plasma PEA levels, but not OEA levels, were increased in 25 morbidly obese individuals, while there were no significant changes in 31 overweight and 21 modestly obese individuals [93]. Kimberly et al. found plasma AEA levels to be positively correlated with body mass index, waist circumference, fasting serum glucose, and abnormal HOMA-IR (*n* = 997 individuals) [94]. Di et al. found that plasma AEA levels were elevated in young adults with higher adiposity and insulin resistance (*n* = 133) [95]. They also found that plasma levels of OEA, PEA, and SEA positively correlated with total CHOL, LDL-C, apoB, and TAGs [95]. Dion et al. found that plasma AEA levels were higher in women with impaired glucose tolerance (IGT, *n* = 9) compared to those with normal glucose tolerance (NGT, *n* = 10) [96]. Plasma OEA, LEA, and PEA were higher in women than men (11 IGT/10 NGT), but did not differ between IGT and NGT individuals [96]. Sugamura et al. found that circulating AEA levels were higher in those with coronary artery disease (CAD, *n* = 20) compared to those without CAD (*n* = 20) [97]. While these studies support the general notion that FAE biosynthesis becomes dysregulated during progression of cardiometabolic diseases, their small sample size and the inconsistency in changes of FAEs make drawing meaningful mechanistic conclusions difficult. Thus, large scale studies are needed to assess whether measurement of plasma or tissue FAE levels can be used to aid important clinical decisions including whether to initiate treatment or the type of treatment that is needed. As importantly, careful mechanistic studies scaling from cultured cells, small animals, and then to humans are needed to provide a deep understanding of how chronic lipid overload alters the expression and activity of FAE biosynthetic enzymes.

## Conclusions

Over the past 25 years, a significant collection of studies have accrued that provide strong support for the notion that biosynthesis of OEA, PEA, and other FAEs, with the resulting activation of FAE receptors like PPARα, GPR119, and GPR55, plays a critical role in limiting the excess accumulation of triglyceride and cholesterol in tissue and circulation, thereby slowing the progression of cardiometabolic diseases. While increases in intracellular free fatty acid levels are clearly an important trigger for increased FAE synthesis, future studies are urgently needed to work out the precise molecular details for this response and why it becomes dysfunctional during development of disease. Furthermore, while the limited number of studies that have used tissue specific deletion of *Napepld* to decrease endogenous FAE levels support the notion that endogenous FAE biosynthesis plays a key role in lipid homeostasis, a definitive understanding of the contribution of the FAE pathway requires studies where FAE biosynthesis is fully ablated. This will require systematic deletion of multiple FAE biosynthetic enzymes or the identification of appropriate inhibitors of these various enzymes. Additionally, while the remarkable effects of therapeutic administration of OEA, PEA, and other FAEs can largely be rationalized by their ability to activate PPARα, GPR119, and GPR55, many key details of their actions are still poorly characterized and require additional studies. Finally, while small scale clinical trials examining the efficacy of therapeutic administration of OEA and PEA against cardiometabolic diseases have been promising, their efficacy needs to be rigorously tested in large-scale randomized clinical trials including for metabolic dysfunction-associated steatotic liver disease and for atherosclerotic cardiovascular disease.

## Key References

- Rodríguez de Fonseca F, Navarro M, Gómez R, Escuredo L, Nava F, Fu J, et al. An anorexic lipid mediator regulated by feeding. Nature. 2001;414(6860):209–12.
  - ○ The paper that first reported that fasting decreased intestinal OEA levels and that re-feeding increased them, as well as that that OEA (and PEA to a lesser extent) inhibited food intake.
- Geurts L, Everard A, Van Hul M, Essaghir A, Duparc T, Matamoros S, et al. Adipose tissue NAPE-PLD controls fat mass development by altering the browning process and gut microbiota. Nat Commun. 2015;6:6495.
  - ○ This paper reports the effect of specifically deleting Napepld in adipose tissue and provides the strongest causative evidence that reduction of endogenous FAE biosynthesis alters triglyceride and cholesterol metabolism.
- Fu J, Oveisi F, Gaetani S, Lin E, Piomelli D. Oleoylethanolamide, an endogenous PPAR-alpha agonist, lowers body weight and hyperlipidemia in obese rats. Neuropharmacology. 2005;48(8):1147–53.
  - ○ The paper that first reported that OEA could protect against obesity and hepatosteatosis in rodents.
- Ostadrahimi A, Khajebishak Y, Moradi F, Payahoo L. The effect of Oleoylethanolamide supplementation on lipid profile, fasting blood sugar and dietary habits in obese people: a randomized double-blind placebo-control trial. BMC Endocr Disord. 2024;24(1):210.
  - ○ This randomized double-blind placebo-controlled clinical trial in adults with obesity showed that daily OEA supplementation significantly reduced their serum triglycerides.
- Rinne P, Guillamat-Prats R, Rami M, Bindila L, Ring L, Lyytikäinen LP, et al. Palmitoylethanolamide Promotes a Proresolving Macrophage Phenotype and Attenuates Atherosclerotic Plaque Formation. Arterioscler Thromb Vasc Biol. 2018;38(11):2562–75.
  - ○ The first paper to report that administration of palmitoylethanolamide definitively reduced atherosclerotic plaque burden in susceptible mice and that palmitoylethanolamide enhanced efferocytosis by cultured macrophages.
- Fu J, Gaetani S, Oveisi F, Lo Verme J, Serrano A, Rodríguez de Fonseca F, et al. Oleylethanolamide regulates feeding and body weight through activation of the nuclear receptor PPAR-α. Nature. 2003;425(6953):90–3.
  - ○ This paper was the first to report that OEA is a potent PPARα agonist and that PPARα is required for OEA to inhibit feeding and to increase fatty acid oxidation in various tissues.
- Overton HA, Babbs AJ, Doel SM, Fyfe MC, Gardner LS, Griffin G, et al. Deorphanization of a G protein-coupled receptor for oleoylethanolamide and its use in the discovery of small-molecule hypophagic agents. Cell Metab. 2006;3(3):167–75.
  - ○ This paper was the first to report that OEA potently activates GPR119 and that other FAEs are also GPR119 agonists.
- Ryberg E, Larsson N, Sjögren S, Hjorth S, Hermansson NO, Leonova J, et al. The orphan receptor GPR55 is a novel cannabinoid receptor. Br J Pharmacol. 2007;152(7):1092–101.
  - ○ This paper provides the most detailed molecular pharmacology of FAEs and synthetic ligands for GPR55 and endocannabinoid receptors 1 and 2 currently available.
- Proulx K, Cota D, Castañeda TR, Tschöp MH, D'Alessio DA, Tso P, et al. Mechanisms of oleoylethanolamide-induced changes in feeding behavior and motor activity. Am J Physiol Regul Integr Comp Physiol. 2005;289(3):R729–37.
  - ○ A comprehensive analysis of various mechanisms that might underlie the satiating effects of OEA that showed these are not dependent on secretion of GLP-1 or other satiation peptide hormones.
- Yang Y, Chen M, Georgeson KE, Harmon CM. Mechanism of oleoylethanolamide on fatty acid uptake in small intestine after food intake and body weight reduction. American Journal of Physiology-Regulatory, Integrative and Comparative Physiology. 2007;292(1):R235–R41.
  - ○ This paper examined the effects of OEA beyond appetite regulation and reported that OEA increases CD36 expression and fatty acid uptake in intestinal cells.
- Igarashi M, Watanabe K, Tsuduki T, Kimura I, Kubota N. NAPE-PLD controls OEA synthesis and fat absorption by regulating lipoprotein synthesis in an in vitro model of intestinal epithelial cells. Faseb j. 2019;33(3):3167–79.
  - ○ A comprehensive analysis of the effects of knocking down NAPE-PLD expression in cultured intestinal epithelial cells that shows that NAPE-PLD activity controls lipoprotein synthesis in these cells.
- Zarrow JE, Alli-Oluwafuyi AM, Youwakim CM, Kim K, Jenkins AN, Suero IC, et al. Small Molecule Activation of NAPE-PLD Enhances Efferocytosis by Macrophages. ACS Chem Biol. 2023;18(8):1891–904.
  - ○ This study shows that deletion or inhibition of NAPE-PLD in macrophages markedly reduces their capacity to carry out efferocytosis, while treatment with small-molecule activation of NAPE-PLD markedly increases their efferocytosis.

## Data Availability

No datasets were generated or analysed during the current study.
